# Predictors of future haemorrhage from cerebral cavernous malformations: a retrospective cohort study

**DOI:** 10.1007/s10143-023-01949-x

**Published:** 2023-02-10

**Authors:** Conor S. Gillespie, Khalifa E. Alnaham, George E. Richardson, Mohammad A. Mustafa, Basel A. Taweel, Abdurrahman I. Islim, Cathal John Hannan, Emmanuel Chavredakis

**Affiliations:** 1 Institute of Systems, Molecular and Integrative Biology, University of Liverpool, Biosciences Building, Crown Street, Liverpool, L69 7BE UK; 2 The Walton Centre NHS Foundation Trust, Liverpool, UK; 3 Academic Health Science Centre, University of Manchester, Manchester, UK

**Keywords:** Cavernoma, Cavernous malformation, Cavernous haemangioma, Familial cavernoma, Haemorrhage, CCM

## Abstract

Cerebral cavernous malformations (CCMs) are commonly diagnosed, with a low reported rate of haemorrhage on long-term follow-up. The identification of factors predictive of future haemorrhage risk would assist in guiding the management of patients with CCM. The aim of this study was to identify variables associated with haemorrhage, and calculate haemorrhage risk in CCM. We conducted a retrospective study of patients diagnosed with a CCM, managed at a specialist tertiary neuroscience centre (2007–2019). The primary outcome was symptomatic haemorrhage, and secondary outcomes were variables associated with increased risk of haemorrhage, using multivariable Cox regression analysis. Included were 545 patients, with 734 confirmed cavernomas. Median age at diagnosis was 47 (interquartile range [IQR] 35–60), with a median follow-up duration after diagnosis of 46 months (IQR 19–85). Of the patients, 15.0% had multiple lesions (*N* = 82/545). Symptomatic presentation was observed in 52.5% of patients (*N* = 286/545). The annual haemorrhage rate was 1.00% per lesion-year (25 events in 2512 lesion-years), and higher in those with symptoms at presentation (1.50% per lesion-year, 22 events vs 0.29%, 3 events, *P* < 0.001). The variables associated with symptomatic haemorrhage were increased size (hazard ratio [HR] 1.04, 95% confidence interval [CI] 1.01–1.07, *P* = 0.004), eloquent location (HR 2.63, 95% CI 1.12–6.16, *P* = 0.026), and symptomatic haemorrhage at presentation (HR 5.37, 95% CI 2.40–11.99, *P* < 0.001). This study demonstrated that CCMs have a low haemorrhage rate. Increased size, eloquent location, and haemorrhage at presentation appear to be predictive of a higher risk of haemorrhage, and could be used to stratify management protocols.

**Supplementary Information:**

The online version contains supplementary material available at 10.1007/s10143-023-01949-x.

## Introduction

Cerebral cavernous malformations (CCMs) are low-flow vascular malformations composed of hamartomatous clusters of thin-walled capillaries, with an incidence of between 0.4 and 1.0% in the general population [1, 2]. The vast majority of patients do not experience any symptoms following diagnosis; however, a subset of CCMs are prone to haemorrhage, with a reported rate of 0.3–6% per patient year [3, 4].

Up to 40% of CCMs are discovered incidentally, and with this figure increasing over time [5], optimal monitoring strategies have become increasingly important [6, 7]; the factors associated with haemorrhage are still yet to be definitively identified. Previous studies have reported having a single lesion, larger size, and infratentorial location as factors associated with a symptomatic haemorrhage [8, 9].

A study with a large sample size, using established definitions, will improve the existing evidence base [10], and assist in validation of the previously identified factors. This would help guide management paradigms in patients with CCM, especially those that present with symptomatic haemorrhage, familial CCM, or those discovered incidentally [11].

## Objectives

The primary objective of the study was to evaluate variables associated with symptomatic haemorrhage risk in a large cohort of patients with CCM. Secondary objectives included determination of annual haemorrhage rate and lesion characteristics. Based on previous studies we hypothesised that single lesion, larger size, and infratentorial location would be associated with increased risk of symptomatic haemorrhage.

## Materials and methods

### Study design, setting, and participants

A single-centre, retrospective cohort study was performed, of all CCMs identified by searching a radiological imaging database for terms related to CCM between 1 January 2007 and 1 January 2019. The study was approved by the local hospital audit committee. Adults ≥18 years diagnosed with at least one CCM, confirmed by a board certified neuroradiologist during the study period, were eligible for inclusion. Patients with any spinal CCMs (excluding patients with coexisting brain CCM), absence of confirmed radiological diagnosis, and incomplete health records/follow-up data were excluded. The study setting was a tertiary neuroscience centre in England, UK, with a catchment area of approximately 3.5 million people. Patients were identified from coding lists from the centre’s radiology department, searching for ‘cavernoma’, ‘CCM’, and ‘cavernous malformation’.

### Terms and definitions

Haemorrhages were classified in accordance with reporting standards from the Angioma Alliance [12], with a symptomatic haemorrhage defined as radiological (CT or MRI) evidence of acute haemorrhage, associated with acute or subacute clinical symptoms. ‘Interval change’ during follow-up was defined as a change in signal intensity on T2-weighted MRI scan, without symptoms suggestive of haemorrhage. Size was measured as maximum diameter including surrounding hemosiderin on T2-weighted, 1.5- to 3-T MRI imaging, as described previously [8]. If multiple intracranial lesions were present, for the per-patient analysis, we used the median diameter of combined cavernoma. If no genetic testing was completed, a familial cerebral cavernous malformation (FCCM) was defined by established criteria as the presence of both: diffuse CCM (five or more) or occurrence of CCM in at least two first-degree family members. In patients with genetic testing available, FCCM was defined as confirmation of one of three genetic mutations known to be associated with FCCM (CCM1, CCM2, and CCM3) [13, 14]. Follow-up duration was defined as the time in months from initial diagnosis until last clinic review with a neurosurgeon.

### Baseline characteristics and management data

Baseline characteristics recorded included age at diagnosis, presentation (symptomatic vs incidental), lobe location, if the CCM was located in an eloquent brain area in the cerebral hemispheres (defined according to the Spetzler-Martin criteria as the sensorimotor cortex, language and visual cortex, hypothalamus and thalamus, internal capsule, brainstem, cerebellar peduncles, and deep cerebellar nuclei) [15], if the CCM was associated with previous cranial irradiation, symptomatic haemorrhage during follow-up, asymptomatic haemorrhage during follow-up (defined as being identified on radiological imaging only without corresponding clinical symptoms), and if data on clinical management such as microsurgical resection, stereotactic radiosurgery (SRS), and the indications for initiating treatment were also identified. Information was recorded and analysed both on a per-patient and on a per-lesion level.

### Study endpoints

The study primary endpoint was symptomatic haemorrhage during follow-up. Patients that presented with symptomatic haemorrhage were considered to have met this endpoint only in the event of a further symptomatic haemorrhage. Secondary endpoints were intervention (with surgery or SRS) or end of follow-up period/discharge from routine monitoring.

### Statistical analysis

Statistical analysis was conducted using IBM SPSS Statistics (Version 27; IBM Corp, Armonk, NY, USA), and graphical representation using R V4.0.2 (ggplot2, survminer, and finalfit packages). Patient characteristics were represented using descriptive statistics, with skewed data represented using median and interquartile range (IQR), and normally distributed data using means and standard deviation (SD). The Chi square test was used to examine statistical differences in outcomes for categorical variables. The Student *t*-test, Mann-Whitney *U* test, or Kruskal-Wallis test was used to examine continuous variables as appropriate. Correlation between baseline variables was evaluated using the Pearson correlation coefficient.

Incidence of haemorrhage rate was calculated at the lesion level. Prognostic factors for symptomatic haemorrhage were delineated using stepwise multivariate proportional hazard regression analysis, incorporating variables with *P* values ≤0.1 on univariate analysis. A *P* value of <0.05 on multivariable analysis was considered significant. Analysis of haemorrhage predictors was completed on both per-patient and per-lesion level, to ascertain any differences between the two groups [16]. We investigated the proportional hazard assumption of both per-patient and per-lesion model by using Schoenfeld residual plots.

## Results

### Baseline characteristics

The baseline, clinical, and radiological characteristics are shown in Table 1. A total of 545 eligible patients with 734 confirmed CCMs were identified. The mean age at diagnosis was 47.3 years of age (SD 15.7), and 50.8% were female (*N* = 277). Four-hundred sixty seven (84.8%) had a single CCM, whilst 82 (15.0%) had multiple. The median number of CCMs per patient with multiple CCMs was 3 (IQR 2–4). A total of 33 patients with 126 CCMs were identified as having FCCM. In total, 11 CCMs were associated with previous radiation treatment (2.0%), with one (0.1%) being associated with previous SRS treatment.

**Table 1:** Baseline characteristics of the cohort of 545 patients with 734 CCM

Baseline characteristics	Value (%) [SD] {IQR}
Total patients	545
Total cavernoma	734
Patients with single cavernoma	463 (85.0)
Patients with multiple cavernoma	82 (15.0)
Male	268 (49.2)
Female	277 (50.8)
Mean age at discovery in years	47.3 [15.7]
Familial (suspected or confirmed^*^)	108 (20.3)
Familial (confirmed only)	97 (18.3)
Symptomatic	Frequency
Yes	286 (52.5)
No	252 (46.2)
Unknown	7 (1.3)
Symptoms	Frequency
Symptomatic haemorrhage	132 (24.0)
Seizure	104 (18.9)
Headache	18 (3.3)
Cranial nerve deficit	8 (1.4)
Limb weakness/paraesthesia	7 (1.2)
Other	16 (2.9)
Location	Frequency
Cortex	411 (56.5)
Subcortex	156 (21.4)
Brainstem	96 (13.1)
Cerebellum	65 (8.9)
Other	6 (0.8)
Infratentorial	161 (22.1)
Lobe	Frequency
Frontal	221 (38.1)
Temporal	145 (19.8)
Parietal	97 (16.7)
Occipital	69 (11.9)
Other	203 (37.5)
Eloquence	Frequency
Eloquent	331 (45.1)
Non-eloquent	403 (54.9)
Size	Median {IQR}
Size in mm	11 {7–15}
Follow-up	Median {IQR}
Clinical follow-up period in months	46 {19–85}
Management strategy	Frequency
Active monitoring	659 (89.8)
Surgery	72 (9.8)
Radiosurgery	4 (0.4)
Haemorrhage rates per year	Per-lesion year
Overall	1.00%
Symptomatic cavernoma	1.50%
Asymptomatic cavernoma	0.29%

*SD* standard deviation, *IQR* interquartile range

^*^Confirmed = confirmed by genetic testing, suspected = suspected but not confirmed with genetic testing

Two-hundred fifty-two (46.2%) patients presented incidentally, with 286 (52.5%) having symptoms at diagnosis. Of these, 104 (18.9%) presented with seizures localising to the site of the CCM, 132 (24.0%) with a symptomatic haemorrhage, and 49 (8.9%) with other symptoms (Table 1).

### Lesion characteristics

Of the 734 CCMs, 411 (56.5%) were located in the cortex, followed by subcortex (21.4%, *N* = 156), brainstem (13.1%, *N* = 96), and cerebellum (8.9%, *N* = 65). Located in an eloquent brain area (*N* = 331) were 45.1%. The median diameter of CCM was 11 mm (IQR 7–15, range 2–65).

### Follow-up period

The median follow-up time was 46 months (IQR 19–85). There were a total of 25 haemorrhages recorded during the follow-up period. Interval change without symptoms of haemorrhage was reported for 32 CCMs. The majority of CCMs were managed conservatively, with 72 (9.1%) being treated surgically and 4 (0.5%) with radiosurgery. The median time to intervention was 9 months following diagnosis (IQR 3–40 months), with 33.3% (*N* = 24/72) being treated within the first 3 months of diagnosis. Twenty-eight patients died during the study period, with five due to CCM haemorrhage specifically during follow-up.

### Haemorrhage rate

**Fig. 1:**
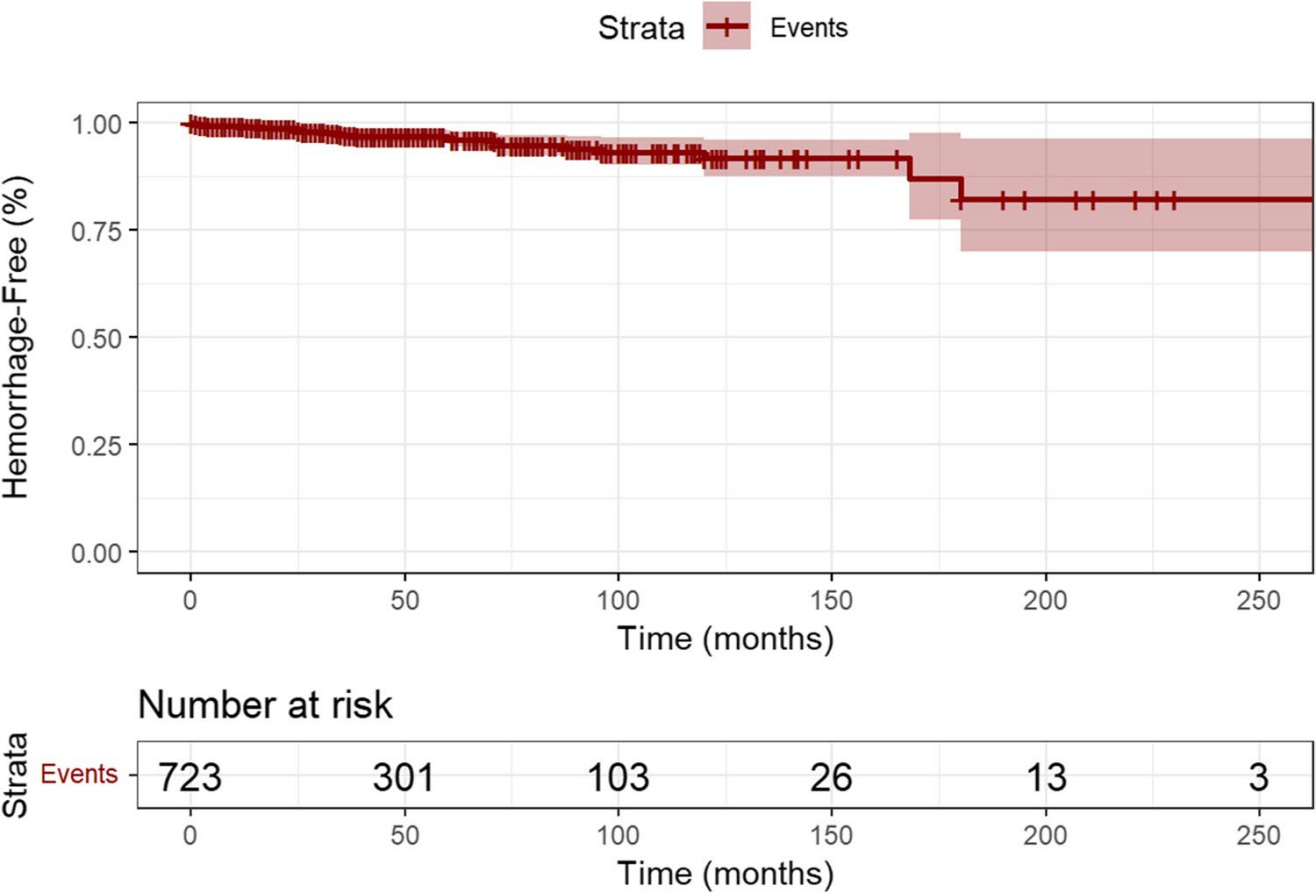
Kaplan-Meier curve demonstrating symptomatic haemorrhage risk in cerebral cavernoma

**Fig. 2:**
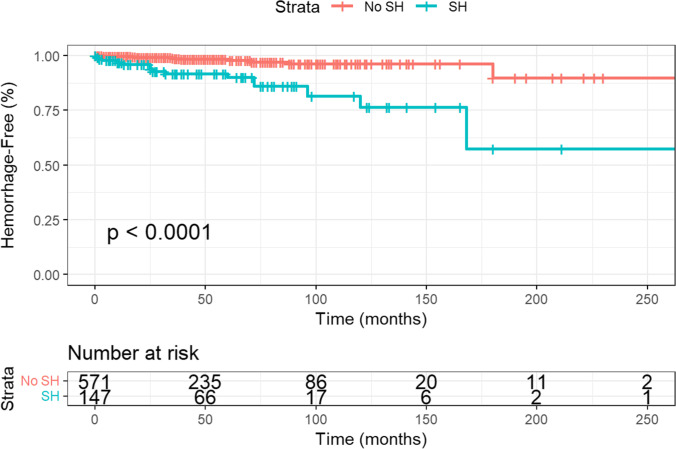
Kaplan-Meier curve demonstrating symptomatic haemorrhage risk in cerebral cavernoma, stratified by symptomatic haemorrhage (SH) as initial presentation

A total of 161 haemorrhages occurring in 148 CCMs were recorded, of which 136 (84.5%) occurred at presentation, and 25 (15.5%) during follow-up (Fig. 1). Including all CCMs, the annual haemorrhage rate was 1.00% per lesion-year (25 events in 2512.25 lesion years). Of the haemorrhages, 20% (*N* = 5) occurred within the first year of presentation, with 80% (*N* = 20) occurring between 12 and 180 months after diagnosis. CCMs presenting with symptoms (seizures, headache, or other symptoms due to haemorrhage at presentation) exhibited a haemorrhage rate of 1.50% per lesion-year (392 CCMs, 22 events in 1464.58 lesion-years), compared to CCMs that presented asymptomatically with a rate of 0.29% (352 CCMs, 3 events in 1047.67 lesion-years, *P* < 0.001) (Fig. 2).

### Factors associated with haemorrhage

**Fig. 3:**
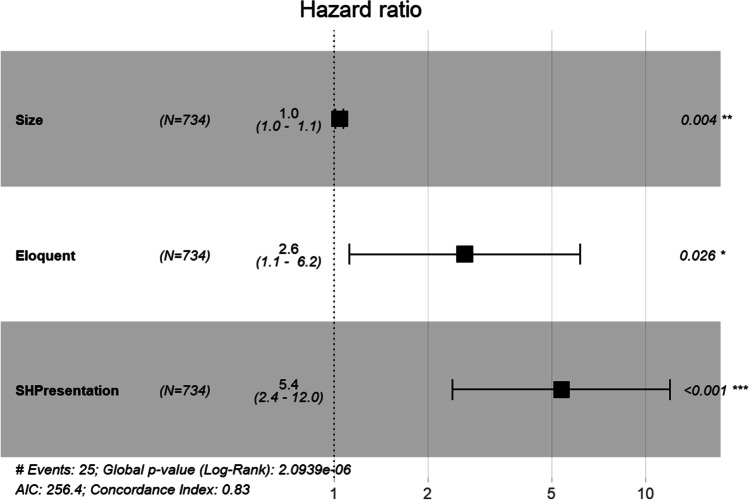
Forest plot of hazard ratios (HR) of factors associated with future haemorrhage on multivariable analysis (*SH* = symptomatic haemorrhage)

The factors associated with haemorrhage are shown in Table 2. On univariable analysis, factors associated with haemorrhage were increasing size (HR 1.05, 95% CI 1.02–1.08, *P* = 0.002), eloquent area (HR 2.60, 95% CI 1.12–6.04, *P* = 0.026), and symptomatic haemorrhage at presentation (HR 5.64, 95% CI 2.53–12.58, *P* < 0.001) (Table 2). On multivariable analysis, the significant factors for haemorrhage were size (HR 1.04, 95% CI 1.01–1.07, *P* = 0.004), eloquent area (HR 2.63, 95% CI 1.12–6.16, *P* = 0.026), and symptomatic haemorrhage at presentation (HR 5.37, 95% CI 2.40–11.99, *P* = 0.001) (Fig. 3). None of the covariates in the Cox regression were significantly time dependent on Schoenfeld residual testing (Supplementary Figures 1 and 2), indicating proportional hazards. A linear relationship was observed with increased cavernoma size and risk of haemorrhage, when using binary size definitions (Table 3).

**Table 2:** Cox regression analysis of variables associated with symptomatic haemorrhage (per-lesion). * = P<0.05

Univariable analysis—per lesion		
Risk factor	Hazard ratio (95% CI)	*P* value
Age	1.00 (0.98–1.03)	0.924
Male sex	1.04 (0.47–2.31)	0.929
Size	1.05 (1.02–1.08)	0.002*
Location (cortex)	1.73 (0.73–4.11)	0.211
Brainstem location	0.32 (0.04–2.39)	0.269
Frontal lobe location	1.28 (0.43–3.80)	0.651
Infratentorial location	0.32 (0.04–2.39)	0.269
Eloquent area	2.60 (1.12–6.04)	0.026*
Familial	0.52 (0.15–1.75)	0.289
XRT associated	3.01 (0.40–22.47)	0.282
Symptomatic haemorrhage presentation	5.64 (2.53–12.58)	<0.001*
Multivariable analysis—per lesion		
Risk factor	Hazard ratio (95% CI)	*P* value
Size	1.04 (1.01–1.07)	0.004*
Eloquent area	2.63 (1.12–6.16)	0.026*
Symptomatic haemorrhage presentation	5.37 (2.40–11.99)	<0.001*

*CI* confidence interval, *XRT* X-ray treatment

**Table 3:** Relationship between size and haemorrhage risk (per-lesion) stratified as a categorical variable. * = P<0.05

Univariable analysis—per lesion		
Size category (*N*)	Hazard ratio (95% CI)	*P* value
<10 mm (290)	NA (reference category)	-
10–19 mm (315)	2.67 (0.87–8.19)	0.088*
20–29 mm (77)	3.01 (0.74–12.2)	0.122
30–39 mm (21)	5.45 (0.98–30.48)	0.053*
>40 mm (13)	11.77 (2.15–64.37)	0.004*
Multivariable analysis—per lesion		
Size category (*N*)	Odds ratio (95% CI)	*P* value
<10 mm (289)	NA (reference category)	-
10–19 mm (313)	2.49 (0.80–7.73)	0.114
20–29 mm (77)	2.79 (0.69–11.4)	0.152
30–39 mm (21)	6.68 (1.19–37.34)	0.031*
>40 mm (12)	8.93 (1.62–49.20)	0.012*
Binary variable (*N*)	Odds ratio (95% CI)	*P* value
≥20 mm (109)	2.20 (0.95–5.14)	0.068
≥25 mm (57)	2.75 (1.03–7.34)	0.044*
≥30 mm (33)	3.90 (1.33–11.41)	0.013*

*CI* confidence interval

### Per-patient analysis

Per-patient analysis is shown in Table 4. On per-patient analysis, the factors that remained associated with haemorrhage in multivariable analysis were eloquent area (HR 3.25, 95% CI 1.42–7.44, *P* = 0.005) and symptomatic haemorrhage at presentation (HR 3.94, 95% CI 1.68–9.21, *P* = 0.002). Size was not significant on per-patient analysis (HR 1.03, 95% CI 1.00–1.06, *P* = 0.108). None of the per-patient covariates in the Cox regression were significantly time dependent on Schoenfeld residual testing (Fig. 3), indicating proportional hazards.

**Table 4:** Cox regression analysis of variables associated with symptomatic haemorrhage (per-patient)

Univariable analysis—per patient		
Risk factor	Hazard ratio (95% CI)	*P* value
Age	1.01 (0.98–1.04)	0.652
Male sex	1.24 (0.44–3.48)	0.678
Size	1.04 (1.00–1.08)	0.088*
Location (cortex)	1.45 (0.44–4.73)	0.540
Brainstem location	0.43 (0.06–3.27)	0.415
Frontal lobe location	1.25 (0.33–4.66)	0.744
Infratentorial location	0.53 (0.12–2.35)	0.402
Eloquent area	8.86 (2.82–27.85)	<0.001*
Familial (suspected or confirmed**)	0.41 (0.09–1.82)	0.239
Familial (confirmed**)	0.22 (0.03–1.67)	0.142
XRT associated	3.74 (0.49–28.62)	0.204
Symptomatic haemorrhage presentation	3.03 (1.10–8.40)	0.033*
Multivariable analysis—per patient		
Risk factor	Odds ratio (95% CI)	*P* value
Size	1.03 (1.00–1.06)	0.108
Eloquent area	3.25 (1.42–7.44)	0.005*
Symptomatic haemorrhage presentation	3.84 (1.68–9.21)	0.027*

*CI* confidence interval, *XRT* X-ray treatment

*Significant

**Confirmed with genetic testing

## Discussion

In this single-centre, retrospective study, examining a 14-year experience of CCMs, we highlight that over 50% of CCMs present with symptoms, with the most common presentations being symptomatic haemorrhage and seizures. The factors associated with symptomatic haemorrhage on multivariable analysis were increasing size, eloquent location, and symptomatic haemorrhage at presentation. Over a median follow-up period of 4 years, the haemorrhage rates were 1.50% per year for CCMs with symptomatic presentation, and 0.29% for those with asymptomatic presentation. We examined symptomatic haemorrhage as our primary outcome, using established clinical-radiological definitions, and reporting standards set by the Angioma Alliance [12]. By employing these criteria and using clinical symptoms, we were able to identify radiological factors associated with CCM haemorrhage. Multiple studies have now demonstrated that location and aetiology are significant factors identifying those CCMs that are at higher risk of bleeding [9, 17]. CCM haemorrhage risk has been noted to be between 14.7 and 17.9% at 5-year follow-up [18], in part due to CCMs being more prone to microhaemorrhage, compared to other cerebrovascular malformations [19].

Our results are similar to a retrospective cohort study conducted by Gomez-Paz, which identified that having a single lesion and increased size (over 10-mm increments) were significantly associated with haemorrhage [8]. We did not identify infratentorial location as being a significant factor associated with haemorrhage, something that was identified in their manuscript, and other papers [20]. A recent multi-centre, prospective study examining risk of CCM haemorrhage on multivariable analysis identified large size, eloquent location, and shorter time since last haemorrhage event to be significantly associated with haemorrhage [21]. Our study identified both large size, eloquent location, and previous symptomatic haemorrhage to be significant, with all of these factors being incorporated into a bleeding risk calculator proposed by the same authors [21].

Our haemorrhage rates of 1.50% per year for symptomatic CCM and 0.29% for asymptomatic CCM are in congruity with Kearns et al., who identified a haemorrhage rate of 2.7% per year for CCMs with a previous symptomatic haemorrhage, and 0.15% per year for those asymptomatic [9]. Kondziolka et al. reported an annual haemorrhage rate of 0.6% per patient year of follow-up of a prospective cohort of 122 patients [22], whilst Porter et al. reported a higher haemorrhage risk of 4.2% per year [23]. However, the cohort size of these studies was 122 and 110 patients, respectively, whilst our cohort size includes 545 patients. The annual incidence of haemorrhage is comparable to a meta-analysis of 25 cavernoma studies that reported a 0.3% haemorrhage risk per year in non-brainstem CCM, and 2.8% in brainstem CCM [24]. The analysis also demonstrated a propensity for rehaemorrhage, a phenomenon that was exhibited in lower rates in our study. Due to disparity in definitions used to define a haemorrhage, we used the Angioma Alliance definition, the same definition employed by Gomez-Paz et al., which may account for the similar haemorrhage rates in these studies [8].

Increasing size has been noted to increase likelihood of haemorrhage in CCM, perhaps due to increased area for occlusion and subsequent recanalization. Eloquent areas including brainstem location have previously been identified as being associated with haemorrhage [25, 26]. It is unclear as to why this was associated with increased haemorrhage rate, but a possible explanation could be that a CCM in close proximity to structures could be at increased risk of causing focal neurological deficits, leading to increased likelihood of causing symptoms, morbidity, and death when a haemorrhage occurs, as opposed to one in a non-eloquent area, which may be more likely to lead to an asymptomatic haemorrhage. Having multiple lesions has not been previously identified as reducing the risk of symptomatic haemorrhage, although the genes implicated in the development of familial CCMs are postulated to lead to the development of lesions with a higher risk of haemorrhage [6, 27, 28]. However, a pooled analysis did not identify multiple lesions to be associated with haemorrhage risk [29]. The authors noted that this could be related to study heterogeneity, with various study designs and haemorrhage definitions employed in the analysis. Consensus reporting of haemorrhage rates, making the distinction between per-patient and per-lesion analysis, and clear definitions are required to ensure pooled analysis of results.

The long-term management practices of CCM are still to be defined, as evidence supporting surgical resection is still conflicting [12]. It is generally accepted to manage incidental CCMs conservatively, with intervention reserved for those CCMs which become symptomatic, although the optimal follow-up period for haemorrhaged CCMs is still to be delineated [30]. The identification of those CCMs that are more prone to haemorrhage based on radiological and clinical factors may be a step forward in optimising and personalising effective management [31]. The Cavernomas: A randomised Effectiveness (CARE) trial aims to compare medical and surgical treatments for symptomatic CCM, and is currently underway [32].

### Limitations of the study

This study is limited by its single-centre, retrospective design. More patients will be symptomatic at presentation due to the study setting being a tertiary neurosurgery centre, which is a highly selected population. In addition, despite having one of the largest population sizes reported in the literature, the haemorrhage rate, and therefore the number of events, was low (*N* = 25). However, the median follow-up time of close to 4 years (46 months) after diagnosis is one of the longer reported follow-up times in the literature, allowing for a more accurate prediction of annual haemorrhage rates than studies with shorter follow-up periods. Secondly, as the majority of the patients in this cohort presented following a symptomatic haemorrhage, and there is no screening programme at our institution, it is not possible to determine if the CCMs experienced a symptomatic haemorrhage, prior to presentation at the department or centre. Therefore, CCM that have had a previous haemorrhage before discovery would not be included in the identification of previous haemorrhage as a risk factor. The inclusion of cavernoma that present with SH (52.5%) in the cohort is therefore also controversial.

Retrospective reviews at tertiary neurosurgical centres may have also led to a degree of selection bias—as management strategies may be favoured towards those delivering surgical treatment, selecting a population [24]. As all CCMs diagnosed within our catchment population were referred to our centre, we identified patients by searching scan registries to include all CCMs, addressing this potential selection bias to include all incidental CCMs, which are more likely to be managed conservatively.

Analysis was conducted primarily on a per-lesion level, and not on a per-patient level. This is because whilst those with multiple CCMs will be affected by per-lesion analysis, employing per-patient analysis increases susceptibility to within-subject correlation. A previously reported paper carried out a sensitivity analysis, identifying that no differences existed in prognostic factors for haemorrhage [8]. We elected to carry out analysis on a per-lesion level due to this. Per-patient analysis theoretically violates the assumptions seen in Cox regression analysis, as every lesion is not seen to be independent of one another; however, per-patient analysis did not violate Cox assumptions in our study. The multiple natures of CCMs may display differential causes, some arising from a familial mechanism, and others de novo. Per-lesion analysis, whilst feasible for single cavernoma, may be less useful for familial or multiple CCM, which may display inherently different characteristics, that change over time. This is an area that requires further research.

The radiological factors identified in the regression analysis allow for risk stratification according to the variables, supported by high patient numbers included in our series. These can be used by clinicians to help categorise haemorrhage risk, and develop patient-focussed management strategies for each CCM, as opposed to each patient. These features have already begun to be utilised in haemorrhage risk calculators [21].

Finally, although our series is very large, the limited number of future haemorrhages is not substantial enough to permit stratified analysis by previous haemorrhage due to lack of statistical power. This limitation can be overcome by using pooled effect models, with the study data providing granularity for future individual patient meta-analysis.

## Conclusions

In our large, retrospective series, CCM commonly present both with symptoms, and as an incidental diagnosis. The haemorrhage rate in the first few years of follow-up is low, with increasing size and eloquent area being associated with haemorrhage risk. A conservative management strategy could be considered for these patients to optimise management.

### Supplementary information

ESM 1
