# Supplementary material for: Predictors of future haemorrhage from cerebral cavernous malformations: a retrospective cohort study
Source: Neurosurg Rev. 2023 Feb 10;46(1):52. doi: 10.1007/s10143-023-01949-x (PMC9918566; doi:10.1007/s10143-023-01949-x)
Supplement: Supplementary file 1 — ESM 1 [file 10143_2023_1949_MOESM1_ESM.docx]

**Supplementary Figures**

**
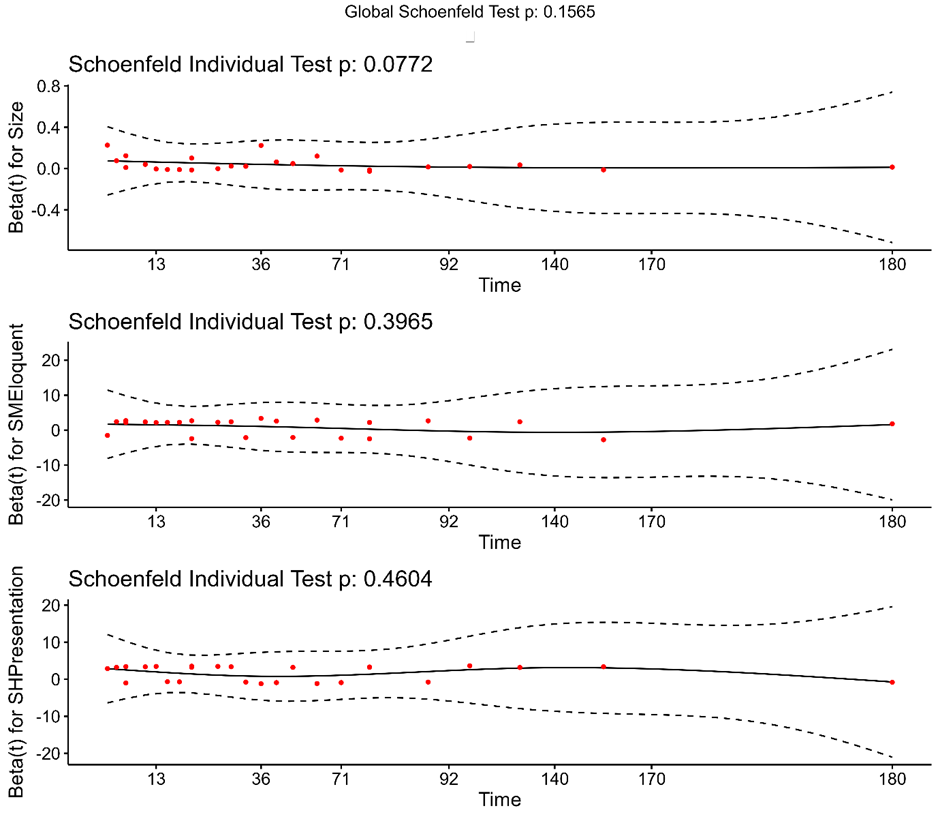
**

Supplementary Figure 1. Schoenfeld residual plot for each of the covariates (per-lesion). The solid line is a smoothing spline fit to the plot, with the dashed lines representing a ± 2-standard-error band around the fit. None of the plots demonstrated a regular pattern with time, and tests were all not statistically significant.


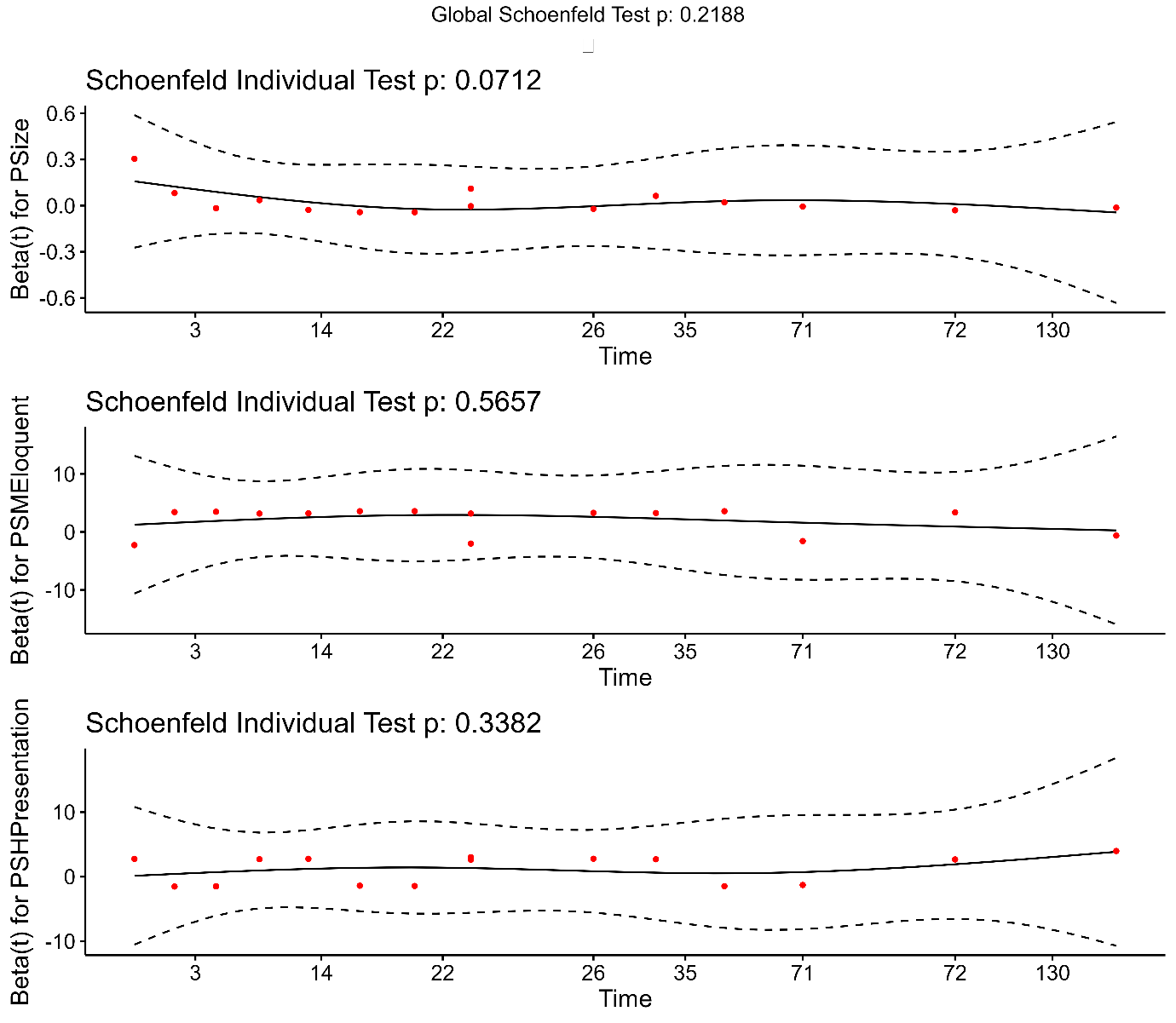


Supplementary Figure 2. Schoenfeld residual plot for each of the covariates (per-patient). The solid line is a smoothing spline fit to the plot, with the dashed lines representing a ± 2-standard-error band around the fit. None of the plots demonstrated a regular pattern with time, and tests were all not statistically significant.
